# Correlation Between Thyroid Hormone Concentrations and Ultrasound Thyroid Volume in Preterm Infants Born Before 33 Weeks of Gestation

**DOI:** 10.3389/fendo.2022.860716

**Published:** 2022-05-10

**Authors:** Aleksandra Mikołajczak, Katarzyna Kufel, Renata Bokiniec

**Affiliations:** ^1^ Neonatal and Intensive Care Department, Institute of Mother and Child, Warsaw, Poland; ^2^ Neonatal and Intensive Care Department, Medical University of Warsaw, Warsaw, Poland

**Keywords:** thyrotropin, thyroxine, preterm infants, ultrasound, thyroid volume

## Abstract

**Objective:**

Thyroid disorders are commonly concomitant with premature birth; however, indications to start therapy remain unclear due to a lack of gestational age (GA)-specific reference ranges. We aimed to evaluate the age-specific thyroid-stimulating hormone (TSH), free thyroxine (FT4) levels and the correlation between TSH and FT4 serum levels and ultrasound thyroid volume in preterm infants.

**Materials and Methods:**

This was an observational, prospective, single-center study of 98 preterm infants born before 33 weeks GA. The infants were divided into the 24-28 weeks and 29-32 weeks GA groups. TSH and FT4 serum levels were measured at two time points: at postnatal age (PNA) 2 weeks and at postmenstrual age (PMA) 32 weeks; the results were compared between groups at two consecutive time points.

**Results:**

There was a statistically significant between-group difference in FT4 concentration. There was a positive correlation between FT4 and GA at both screening times. FT4 in the 24-28 weeks GA group was significantly lower than in the 29-32 weeks GA group. The mean (standard deviation [SD]) FT4 at PNA 2 weeks was 11.72 ± 2.16 pmol/l for the 24-28 weeks GA group vs. 13.33 ± 1.80 pmol/l for the 29-32 weeks GA group (p<0.001). The mean (SD) FT4 at PMA 32 weeks was 11.96 ± 1.98 pmo/l for the 24-28 weeks GA group vs. 13.33 ± 1.80 pmol/l for the 29-32 weeks GA group (p=0.001). Our results reflect a slow and gradual upward trend of FT4 in the 24-28 weeks GA. It is of interest that the correlation between thyroid volume and FT4 was statistically significant (rho=0.25, p=0.019) for all studied preterm infants. The correlation between thyroid volume and weight was statistically significant for the entire study group (rho=0.37, p<0.001). We did not find statistically significant differences in TSH and FT4 values between consecutive time points at 24-28 weeks GA. The thyroid volume was not significantly different between both groups. The total thyroid volume was 0.26 vs. 0.27 ml for the 24-28 and 29-32 weeks GA groups, respectively.

**Conclusion:**

The results of this study indicate that preterm infants require lower FT4 values depending on GA. Moreover, ultrasound thyroid imaging may facilitate the evaluation of questionable thyroid disorders.

## Introduction

Thyroid hormones are crucial for metabolism and thermogenesis and thus for the normal development of the central nervous system in fetuses and infants. Congenital hypothyroidism (CH), the most common endocrinological disorder, is a preventable cause of neurodevelopmental disability. Early diagnosis and treatment of CH can help prevent neurodevelopmental impairment and improve long-term outcomes (1).

The newborn screening test (NST) for CH has recently been improved through the adoption of a lowered thyroid-stimulating hormone (TSH) level as a basis for the diagnosis of CH, is one of the most valuable tools for preventing intellectual disability (2). The increasing incidence of positive screening tests for hypothyroidism performed in all infants, including preterm infants, has given new, greater importance to the issue.

Thyroid disorders are commonly associated with prematurity (3, 4). The higher incidence of infant hypothyroidism might be associated with an increased survival rate of preterm neonates, along with changes in the NST. The prevalence of transient hypothyroidism in preterm infants below 1500 g at birth is 14-fold higher than that in mature infants (4). Preterm infants are more vulnerable to thyroid dysfunction for many reasons, including hypothalamic-pituitary immaturity, serious neonatal illness, impaired synthesis and metabolism of thyroid hormones, and the use of drugs (e.g., dopamine or steroids) (2–6).

The thyroid response to the action of drugs administered to preterm infants has not been sufficiently studied. Dopamine, an adrenergic neurotransmitter that inhibits TSH secretion, also suppresses thyroxine (T4) secretion. However, the discontinuation of dopamine administration results in an immediate increase in T4 levels (4, 7, 8). In contrast to dopamine, dobutamine does not influence the thyroid hormone profile (8). Steroid administration can diminish the TSH response and peripheral conversion of T4 to T3 (4, 7). Studies conducted by Arai et al. confirmed a rapid elevation of free thyroxine (FT4) serum levels in samples collected two days after steroid withdrawal (7).

However, thyroid disorders in preterm infants remain unrecognized. This is likely due to a delay in the elevation of TSH concentration after delivery (1, 5). TSH concentrations tend to rise between the second and sixth weeks of life, although the exact time of TSH elevation can vary. To cope with the resultant problem, European and American guidelines recommend a repeated screening test for hypothyroidism in preterm infants. According to European guidelines, rescreening for CH should be performed in all preterm infants two weeks after the first screening or in the second week of life. The American Academy of Pediatrics advocates repetition between the second and sixth weeks of life (4). However, there is no universal agreement on the optimal timing of blood sample collection for hypothyroidism assessment in preterm infants. Consequently, novel methods for assessing thyroid function are being sought. Thyroid ultrasound imaging might perform this function.

Ultrasound might prove helpful in determining the anatomy and function of the thyroid gland in infants (9). In particular, ultrasound-assessed thyroid volume in neonates may help detect thyroid dysfunction. Ultrasound examination also provides information on the location and structure of the gland. Although ultrasonography helps reveal abnormalities in the location of the thyroid gland, it is not sufficient to diagnose its hypertrophy or hypoplasia (10). Given the simplicity, non-invasive character, and repeatability of the ultrasound examination, we strongly advocate that it should be included as a standard examination in the initial diagnosis of neonates with abnormal screening test results for hypothyroidism. Our opinion is consistent with the guidelines on CH from the European Society for Pediatric Endocrinology, which recommends the performance of imaging studies to determine a specific etiology (11).

There are no currently unavailable normative thyroid ultrasound data for neonates, although significant differences in the mean thyroid volume were detected on ultrasound according to gestational age (GA) (12). The determination of normative ultrasound thyroid volume values could allow the objective identification of a gland as normal, small, or enlarged. Combined with age-specific thyroid hormone reference levels, these reference values could be used to help neonatologists interpret thyroid hormone and ultrasound results in neonates with greater accuracy.

This study aimed to determine the specific GA TSH and FT4 levels in preterm infants born at <32 weeks GA and determine the distribution of FT4 values. Therefore, the preterm infants in our study were divided into two groups: those born between 24-28 and 29-32 weeks GA. In addition, we sought additional methods to assess the physiology of the development and dysfunction of the thyroid gland in preterm infants. We also attempted to determine whether the concentrations of thyrotropic hormone and FT4 correlate with the ultrasound thyroid volume and GA, thus decreasing the percentage of false-negative cases. We further attempted to determine the optimal chronological age that correlates with delayed TSH elevation.

## Subjects and Methods

### Subjects and Eligibility Criteria

This was an observational, prospective, single-center, population-based cohort study. Preterm infants born between 24 and 32 weeks of gestation who were born in or transferred to the Neonatal and Intensive Care Department of the Medical University of Warsaw in Princess Anna Mazowiecka Hospital (Poland) were recruited for this study. The patients’ GA was estimated using ultrasonography. The study was initiated in January 2020, and patients were recruited between 2020 and 2021. Ninety-eight participants were recruited within 7 days of birth.

**Figure 1:**
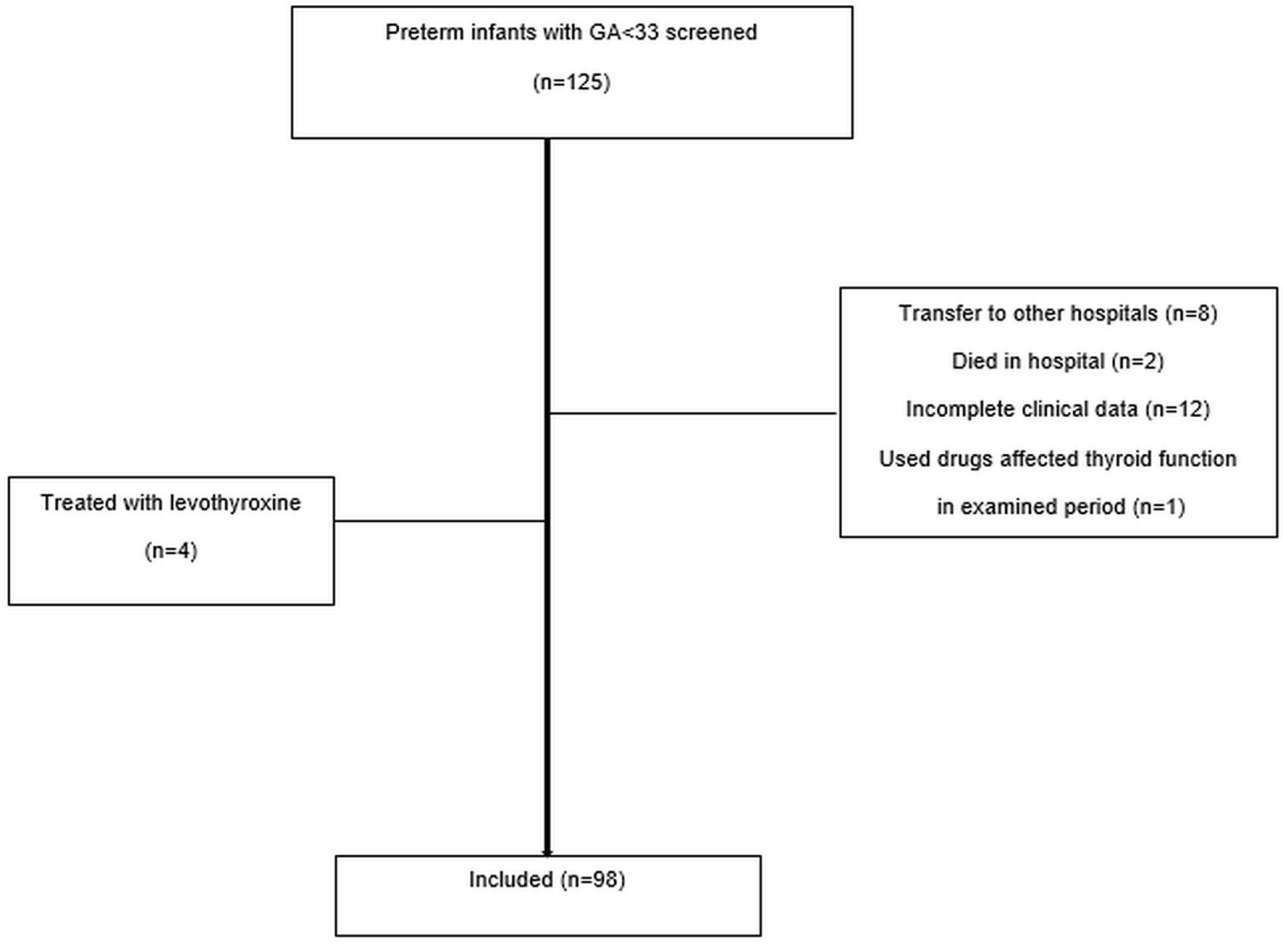
Flow chart of distribution of preterm infants screened.

The inclusion flowchart is presented in **Figure 1**. The eligibility of prospective patients was determined by recruiting physicians familiar with the study protocol. Subjects with the following conditions were excluded from the study: preterm delivery <23 or >32 weeks GA; major congenital abnormalities; administration of medications such as steroids or vasopressors such as dopamine (up to 12 hours after the end of treatment), positive maternal thyrotropin antibodies, thyroid disease of mothers treated with antithyroid drugs or amiodarone, and lack of parental consent.

### Clinical Variable Collection

Maternal medical history included antenatal steroids, maternal diabetes mellitus, and thyroid disorders. The perinatal data included GA, body weight at birth, sex, z-score of birth weight, 1- and 5-min Apgar scores, and delivery method. Clinical data, such as sepsis, necrotizing enterocolitis (NEC), retinopathy of prematurity (ROP), postnatal steroid therapy, dopamine administration, and invasive or non-invasive ventilation, were obtained at discharge. Thyroid function data, including postnatal age (PNA), serum TSH concentration, serum FT4 concentration, and ultrasound thyroid volumes of all recruited preterm infants, were collected prospectively.

To avoid a surge in TSH after birth, blood samples were collected at PNA 2 weeks, and the blood TSH and FT4 levels were measured at 32 postmenstrual age (PMA). The ultrasound thyroid volume was performed at PMA 32 weeks. The baseline data were collected in the hospital electronic database.

### Grouping of the Subjects

The study included 98 infants divided into two groups: those born at 24–28 weeks GA and those born at 29–32 weeks GA. In the 24–28 weeks GA group, FT4 and TSH concentrations were evaluated at 14–21 days of life and PMA 32 weeks. In the 29–32 weeks GA group, FT4 and TSH concentrations were measured at PMA 32 weeks (after 14-21 days of life, equivalent to PMA 32 weeks).

### Detection Methods

Blood samples (1 ml) were collected and examined in the hospital laboratory to measure the levels of serum thyroid hormones during other routine blood tests. The immunofluorescence assays of Architect i1000SR of the Abbott Diagnostic test were used to detect TSH and FT4 levels in serum samples.

Ultrasound evaluation of the neonate thyroid was performed using a Philips HD XE system. (Philips Healthcare, Eindhoven, the Netherlands). One observer scanned the babies at the bedside using a portable scanner. The thyroid gland was measured using a linear array transducer with a high-frequency probe (L7-15 MHz). The volume of the thyroid gland was calculated using the formula for a prolate ellipsoid, where thyroid volume = length × breadth × depth × π/6 (0.52). The total thyroid volume was calculated as the sum of the volume of the individual lobes, disregarding the volume of the isthmus, since this is very low in normal neonates (13).

### Primary Outcomes

The primary outcomes were as follows: **(**a) assessment of the thyroid function and volume depending on the gestation age; (b) the determination of FT4 and TSH values at 14–21 days of life and at PMA 32 weeks in preterm infants; (c) the determination of the ultrasound thyroid volume at PMA 32 weeks in preterm infants; and (d) the evaluation of correlations between circulating thyroid hormone concentrations and thyroid volume.

### Secondary Outcomes

The secondary outcomes were as follows: (a) the comparison of changes in FT4 evaluated at 14-21 days of life (PNA 2 weeks) and PMA 32 weeks in the 24-28 weeks GA group; (b) the comparison of changes in TSH evaluated at 14-21 days of life (PNA 2 weeks) and PMA 32 weeks in the 24-28 weeks GA group; (c) the comparison of changes in FT4 and TSH evaluated at 14-21 days of life (PNA 2 weeks) between the 24-28 and 29-32 weeks GA groups; (d) the analysis of TSH values over time (to determine the optimal time for TSH measurement); (e) the evaluation of the correlation between thyroid volume and circulating thyroid hormone concentrations with body mass at PMA 32 weeks; and (f) the assessment of the influence of morbidity and drugs administered to neonates on thyroid volume and function.

### Ethical Approval

The study was registered in the Clinical Trials Registry (Registration No. NCT04208503; protocol version 07.01.2020) and approved by the Bioethical Committee of the Medical University of Warsaw (KB44/2019). The participants’ caregivers were informed about the study’s methods and purpose. Written informed consent was obtained from all the participants.

### Statistical Analysis

Statistical analysis was carried out using the R package, version 4.0.5. Nominal variables are presented as n (%), continuous variables as means ± standard deviation (SD), or median (quartile [Q]1, Q3), depending on the data distribution. The data’s normality was validated using the Shapiro-Wilk test and based on skewness and kurtosis values. Groups were compared using the chi-square test or Fisher’s exact test for nominal variables and with Welsch t-test or Mann–Whitney U test for continuous variables, as appropriate. The means and median differences (MD) were calculated, including the 95% confidence interval (CI). Paired comparisons were made using the paired t-test or Wilcoxon test. The correlation analysis was performed using Spearman’s correlation coefficient. Additionally, the agreement between double measurements of thyroid volume for the 32^nd^ week was assessed using the interclass correlation coefficient.

## Results

A total of 125 preterm infants were screened. The final sample included 98 preterm infants after the exclusion of four subjects due to the diagnosis of thyroid disorders, the death of two in the postnatal period, the transfer of eight to another hospital, and one due to the administration of drugs that affected thyroid function, and 12 due to incomplete clinical data. Nine patients were small for GA (SGA), and 26 were born from multiple pregnancies. Our analysis included 37 males (37.8%) and 61 females (62.2%). The mean (SD) GA was 28.26( ± 2.29) weeks, and the mean birth weight (BW) was 1226.02( ± 381.57) g. Among the 98 preterm infants, there were 49 (16 male, 33 female) in the 24-28 weeks GA group and 49 (21 male; 28 female) in the 29-32 weeks GA group. The overall group characteristics are presented in **Table 1**.

**Table 1:** Demographic characteristics of the study cohort.

	24-28 weeks GA	29-32 weeks GA	p-value
n	49	49	
Sex, female, n (%)	33 (67.3)	28 (57.1)	NS
Model of delivery, n (%)			
Caesarean section	31 (63.3)	33 (67.3)	NS
Natural delivery	18 (36.7)	16 (32.7)	NS
GA	26.33 ± 1.39	30.18 ± 1.03	<0.001
SGA/AGA/LGA preterm infants, n (%)			
SGA	4 (8.2)	5 (10.2)	NS
AGA	40 (81.6)	41(83.7)	NS
LGA	5 (10.2)	3 (6.1)	NS
Birth weight, g, mean ± SD	954.69 ± 222.39	1 497.35 ± 307.35	<0.001
Birth weight at 32PMA, g, mean ± SD	1 419.57 ± 235.14	1 556.73 ± 281.49	0.010
Apgar score, 1 min, median (Q1;Q3)	6.00 (5.00;7.00)	7.00 (6.00;8.00)	0.002
Apgar score 5 min, median (Q1;Q3)	7.00 (7.00;8.00)	8.00 (7.00;9.00)	<0.001
Singleton or multiplets, n (%)			
Singleton	41 (83.7)	32 (65.3)	NS
Multiplets	8 (16.3)	17 (34.7)	NS
Prenatal Steroid, n (%)	39 (79.6)	34 (69.4)	NS
Postnatal Steroid, n (%)	12 (24.5)	0 (0.0)	<0.001
Dopamine therapy, n (%)	7 (14.3)	0 (0.0)	0.012
CPAP, n (%)	49 (100.0)	46 (93.9)	NS
Mechanical ventilation, n (%)	35 (71.4)	16 (32.7)	<0.001
Necrotizing enterocolitis, n (%)	0 (0.0)	2 (4.1)	NS
Neonatal Sepsis, n (%)	6 (12.2)	1 (2.0)	NS
ROP, n (%)	4 (8.2)	0 (0.0)	NS
Mothers treated with levothyroxine, n (%)	9 (18.4)	11 (22.4)	NS
Antibiotics, n (%)	18 (36.7)	0 (0.0)	<0.001

AGA, appropriate for gestational age; CPAP, continuous positive pressure; LGA, large for gestational age. Groups were compared using the chi-square test or Fisher’s exact test for nominal variables and the t-test or Mann–Whitney U test for continuous variables; NS, non-statistical.

There were statistically significant between-group differences regarding GA, BW, 1- and 5-min Apgar scores, postnatal steroid and dopamine therapy use, mechanical ventilation frequency, and antibiotics administration (p<0.001). We did not identify statistically significant between-group differences regarding the frequency of CPAP(continuous positive airway pressure), NEC, ROP, and prenatal steroid use.

There was a statistically significant difference in FT4 concentration between the groups. The FT4 in the 24-28 weeks GA group was significantly lower than in the 29-32 weeks GA group. The mean (SD) FT4 at PNA 2 weeks was 11.72 ± 2.16 pmol/l for the 24-28 weeks GA group vs. 13.33 ± 1.80 pmol/l for the 29-32 weeks GA group, MD = -1.61, 95% CI [-2.49, -0.74], p<0.001; and the mean (SD) FT4 at PMA 32 weeks was 11.96 ± 1.98 pmo/l for the 24-28 weeks GA group vs. 13.33 ± 1.80 pmol/l for the 29-32 weeks GA group, MD=-1.37, 95% CI [-2.13, 0.61], p=0.001.

No statistically significant between-group differences were found in the TSH values at PNA 2 weeks and PMA 32 weeks. The median (Q1, Q3) TSH at PNA 2 weeks was 2.55 (1.53, 4.36) mU/l for the 24-28 weeks GA group vs. 2.42 (1.45, 3.22) mU/l for the 29-32 weeks GA group, p=0.650; at PMA 32 weeks the median (Q1, Q3) TSH was 2.55 (1.53, 4.36) mU/l for the 24-28 weeks GA group and 2.42 (1.45, 3.22) mU/l for the 29-32 weeks GA group, p=0.457 (**Table 2**).

**Table 2:** Thyroid parameters between groups categorized by GA.

	24-28 weeks GA	29-32 weeks GA	MD (95% CI)	p-value
TSH – PMA 32 weeks	2.13 (1.41; 3.12)	2.42 (1.45; 3.22)	-0.29 (-0.82; 0.38)	NS
FT4 – PMA 32 weeks	11.96 ± 1.98	13.33 ± 1.80	-1.37 (-2.13; -0.61)	0.001
TSH – PNA 2 weeks	2.55 (1.53; 4.36)	2.42 (1.45; 3.22)	0.13 (-0.59; 0.93)	NS
FT4 – PNA 2 weeks	11.72 ± 2.16	13.33 ± 1.80	-1.61 (-2.49; -0.74)	<0.001
Volume L – PMA 32 weeks	0.14 (0.11; 0.16)	0.13 (0.11; 0.17)	0.01 (-0.02; 0.02)	NS
Volume R – PMA 32 weeks	0.13 (0.11; 0.15)	0.14 (0.12; 0.19)	-0.01 (-0.03;0.01)	NS
Difference (R minus L)	-0.01 (-0.02; 0.01)	0.00 (-0.02; 0.02)	-0.01 (-0.02;0.002)	NS
Total Thyroid volume-PMA 32 weeks	0.26 (0.22; 0.31)	0.27 (0.24; 0.34)	-0.01 (-0.05;0.02)	NS

TG, thyroid gland; R, right; L, left. Data are presented as median (Q1, Q3) or mean ± SD. Groups were compared using the t-test or Mann–Whitney U test. NS, non-statistical.

Thyroid volume was not significantly different between the two groups. The median of total thyroid volume performed at PMA 32 weeks for the 24-28 weeks GA group and for the 29-32weeks GA group was 0.26 (0.22, 0.31) and 0.27 (0.24, 0.34) ml, respectively; p=0.318 (**Table 2**). There were no statistically significant differences between the two consecutive screening tests for FT4 and TSH in the 24-28 weeks GA group (**Table 3**).

**Table 3:** Comparison of TSH and FT4 according to PNA 2 weeks and PMA 32 weeks in the 24-28 weeks GA group.

	PNA 2 weeks	PMA 32 weeks	MD (95% CI)	p-value
n	49	49		
TSH (mU/l)	2.55 (1.53; 4.36)	2.13 (1.41; 3.12)	-0.42 (-1.23; 0.21)	0.218
FT4 (pmol/l)	11.72 ± 2.16	11.96 ± 1.98	0.24 (-0.65; 1.43)	0.432

Data are presented as median (Q1, Q3) or mean ± SD. The groups were compared using the t-test or Mann–Whitney U test.

The correlation between thyroid volume, FT4 and TSH concentration level, weight, and GA were assessed in all studied preterm infants in the 24-28 and 29-32 weeks GA groups. The correlation between the thyroid volume and weight was statistically significant for the whole study group (rho=0.37, p<0.001) as well as for the 29-32 weeks GA group (rho = 0.55, p < 0.001). Overall, the correlation between thyroid volume and FT4 was statistically significant (rho = 0.25, p = 0.019) for all studied preterm infants at a PNA of 2 weeks. Similarly, there was a positive correlation between FT4 and GA at both screening times at a PNA of 2 weeks (rho = 0.42, p<0.001) and at PMA 32 weeks (rho=0.35, p<0.001). No statistically significant correlation was found for the 24-28 weeks GA group between the thyroid volume and GA, nor for FT4 and TSH. There was an inverse correlation between thyroid volume and TSH concentration level in the 29-32 weeks GA group (rho=-0.34, p=0.018).

These results indicate that factors such as the out-of-the-schedule-blood-test-time administration of postnatal steroids and dopamine did not significantly affect the thyroid parameters in any way in the 24-28 weeks GA group. They were not affected by sepsis, ROP, or NEC. The intra-observer variation was calculated based on 20 sonographic measurements to be 5.6 ± 19.2%, MD=0,87, 95% CI [0.70-0.95].

## Discussion

Preterm infants have a higher risk of thyroid dysfunction than mature infants. In particular, preterm infants show an atypical form of hypothyroidism, which presents a challenge in distinguishing cases of CH and interpreting NST (1, 2, 14). Moreover, there are no reliable standard thyroid hormone reference values for preterm infants. Hence, neonatologists are increasingly aware of the challenging importance of diagnosing and treating thyroid dysfunction in preterm infants. This study investigated the association between thyroid hormone levels and thyroid volume in preterm infants and allowed us to obtain FT4 and TSH concentration values in preterm infants born before 33 weeks GA and evaluate the ultrasound thyroid volume at PMA 32 weeks.

Thyroid disorders in preterm infants include a unique form of hypothyroidism characterized by transient hypothyroxinemia of prematurity (THOP) or delayed TSH elevation and hyperthyrotropinemia despite low serum FT4/T4 (1, 2, 15). THOP is defined as a temporary reduction in FT4 values due to a blunted postnatal increase with no increase in TSH values, which may last from six to eight weeks (4). Hypothyroxinemia of prematurity is a well-described condition. Its incidence in preterm infants varies between 35% and 85% (15, 16). The reported wide discrepancy in the frequency of THOP diagnosis may reflect the lack of consensus about the FT4 level that constitutes the “low” for that specific GA in preterm infants.

**Figure 2:**
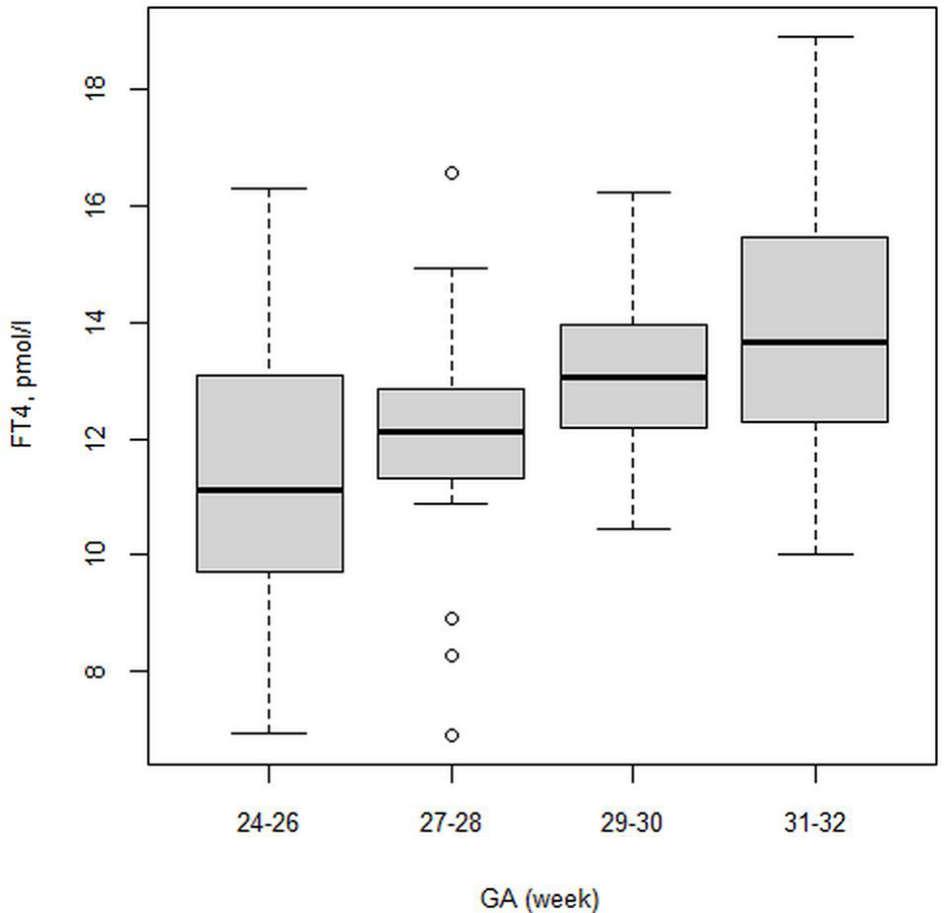
Boxplot showing the distribution of FT4 (pmol/l) level.

**Figure 3:**
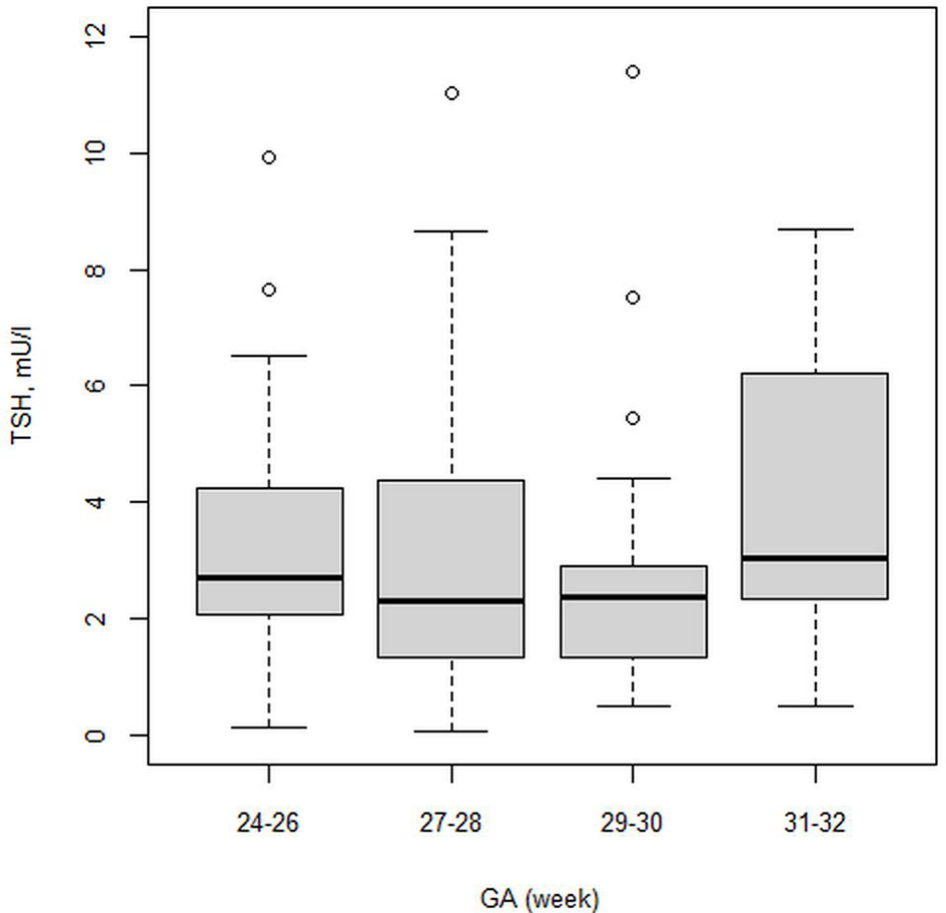
Boxplot showing the distribution of TSH (mU/l) level.

The first finding of our study was that the FT4 serum level positively correlated with GA in the whole study group (rho=0.42; p<0,001 at PNA 2 weeks; rho=0.35; p<0.001 at PMA 32 weeks). We confirmed statistically significant differences in the FT4 serum level (p<0,001) between the 24-28 29-32 weeks GA groups. FT4 levels at PNA 2 weeks were 11.59 and 13.29 pmol/l for the 24-28 and 29-32 weeks GA groups, respectively. Despite this, the FT4 serum levels in the 24-28 weeks GA group compared to those at PNA 2 weeks and PMA 32 weeks were not statistically significant, while FT4 showed a gradual upward trend. We observed higher serum FT4 levels at PMA 32 weeks than at PNA 2 weeks. **Tables 4**, **5** show the 5^th^-95^th^ percentiles for FT4 and TSH at PNA 2 weeks. **Figures 2**, **3** show the distribution of FT4 and TSH according to the GA.

**Table 4:** Distribution of FT4 (pmol/l) values in preterm infants born <33 weeks GA, measured at 2 weeks PNA.

GA (weeks)	5p	10p	25p	50p	75p	90p	95p
24-26 (n=19)	8.129	8.772	9.73	11.11	13.1	14.248	14.951
27-28 (n=20)	8.211	8.856	11.426	12.12	12.855	13.336	15.003
29-30 (n=33)	10.898	11.068	12.2	13.05	13.97	14.73	15.148
31-32 (n=16)	10.988	11.315	12.325	13.64	15.315	17.15	18.2425

p, percentile.

**Table 5:** Distribution of TSH (mU/l) values in preterm infants born <33 weeks GA, measured at 2 weeks PNA.

	5p	10p	25p	50p	75	90	95
24-26 (n=19)	0.7795	1.222	2.0865	2.727	4.241	6.7402	7.8735
27-28 (n=20)	0.51275	0.5998	1.405	2.3135	4.29625	6.7303	8.7682
29-30 (n=33)	0.7994	0.8784	1.33	2.374	2.93	4.2384	6.278
31-32 (n=16)	0.68625	0.829	2.35	3.048	5.8415	10.9275	13.7055

**Figure 4:**
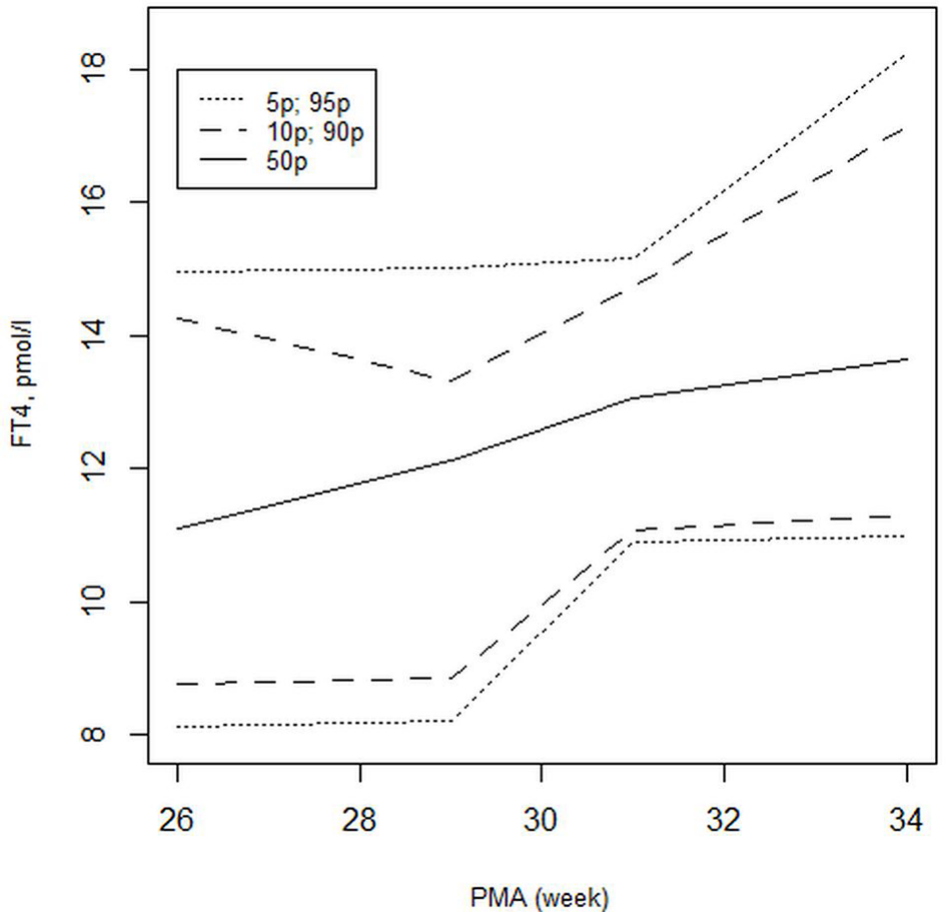
Distribution of FT4 (pmol/l) values presented as the 5th to 95th percentiles for FT4 in preterm infants born with GA <33 weeks at PNA 2 weeks.

Our findings reflect changes in the postnatal elevation of the serum FT4 level according to GA (**Figure 4**); these findings were similar to those reported by Williams et al.—the latter showed that FT4 elevation was attenuated in the 28-30 weeks GA group and was further attenuated in the 23-27 weeks GA group (15, 17, 18) compared with more mature preterm infants. The slow and gradual upward trend in our results of FT4 in the 24-28 weeks GA group might be related to the slow development and maturation of the hypothalamic-pituitary-thyroid axis (19). However, our results differed from Williams’ reported data (18); the values of FT4 at PNA 2 weeks obtained in this study were lower than those in the above-mentioned studies for each GA, but were close to the data reported by Kilchemmann (17). The results obtained by Kilchemmann et al. revealed that the 50^th^ percentiles of FT4 were 0.95 ng/dl [11.19 pmol/l], 1.02 ng/dl [12.75 pmol/l], and 1.11ng/dl [13.86 pmol/l] in the 23-27, 28-30, and 31-34 weeks GA groups, respectively (17). Similarly, an increase in the level of thyroid hormones with increasing GA and PMA was also confirmed by Oh et al. (2). Based on the T3 serum level analysis and the conversion of FT4 to T3, the authors suggested that this increase might be related to the physiological maturation of the thyroid gland or the hypothalamic-pituitary-thyroid axis (2).

The second finding of our study was the determination of serum TSH levels; we observed no statistically significant between-group differences and no correlation with GA. This was similar to the results obtained by Imamoglu et al. (2015), who also did not observe any correlation between TSH levels and GA (20). As a final step in the comprehensive analysis of thyroid function, we estimated the ultrasound thyroid volume. Thyroid ultrasound is one of the most useful, non-invasive imaging tools that may determine the presence or absence of the thyroid tissue in its normal position, show the gland’s overall morphology, and the presence of hemiagenesis or athyreosis. It is important to objectively assess the gland as normal, small, or enlarged by referring to the nomograms of the study population. Our study showed that the thyroid volume was positively correlated with weight in the 29-32 weeks GA group (rho=0.55; p<0.001) and in the whole study group (rho=0.25; p<0.001). Additionally, thyroid volume was positively correlated with FT4 at PNA 2 weeks (rho=0.25; p=0.019). These results are consistent with Ares’s published data, where FT4 values were linearly correlated with the postnatal and postmenstrual age, and FT4 levels were also correlated with the thyroid gland volume (p<0.05) (21).

In contrast, TSH serum levels were negatively correlated with thyroid volume (rho=-0.34; p=0.018). In this study, we determined the thyroid volume at PMA 32 weeks. The median thyroid volume for the 24-28 and 29-32 weeks GA groups were 0.26 (0.22, 0.31) and 0.27 (0.24, 0.34) ml, respectively. In their study of 57 preterm infants with a mean GA of 28.9 weeks and BW of 1181 g, Khan et al. showed serial ultrasound mean thyroid volumes ranging from 0.185-0.237 ml obtained between 13 and 64 days of life (22). These results are consistent with those of our study. In contrast, the results presented in our study were lower than those reported by Kurtoglu et al. The latter were 0.4 and 0.5 ml for preterm infants from the 25-28 and 29-32 weeks GA groups, respectively (12). Further studies are necessary to create nomograms for thyroid volume related to the GA of preterm infants.

During data collection, four subjects were diagnosed with thyroid disorders, and each received L-thyroxine treatment. Ultrasound imaging revealed hemiagenesis of the left lobe in two participants, which was predictive of thyroid disorders. We detected any abnormalities in the other two infants. Follow-up serum testing revealed that in one patient, levothyroxine treatment was discontinued after 1 year. In the whole study group, ultrasound of the thyroid gland revealed the presence of colloid follicles in the form of small 1–3-mm cysts. There were no other related abnormalities.

We would like to point out that an ultrasound examination of the thyroid gland is not required for diagnosis and possible treatment decision but in “doubt cases” it may constitute an additional reasonable factor contributing to a pragmatic conclusion. Given new technical possibilities, the use of good quality ultrasound probes of different sizes may improve diagnostic accuracy. The ultrasound evaluation may help neonatologists to take the decision about instituting a substitutive treatment. It is particularly important in the evaluation of infants referred with TSH elevation.

We have to highlight that a normal thyroid gland does not exclude the necessity of treatment.

The above examples point to the usefulness of the ultrasound examination.

Based on our results, we cannot confirm the impact of morbidities such as sepsis, NEC, ROP, or mechanical ventilation on thyroid hormone levels or thyroid volume. Similarly, Hemmati et al. did not find any significant changes in TSH and FT4 levels in critically ill neonates; Hemmati et al. showed transient elevation of TSH during recovery from the illness, but the FT3 and FT4 levels were not affected (23). In addition, our results showed no significant difference in FT4 and TSH values in preterm infants treated with dopamine or postnatal steroid administration in the out-of-schedule blood test time. Studies that investigated the use of drugs affecting thyroid hormone levels, such as glucocorticoids and dopaminergic agents, confirmed that these drugs may diminish TSH secretion and FT4 serum levels (6, 7). Nonetheless, after discontinuation of dopamine, a quick reversal was observed in this study. Dobutamine does not influence the thyroid hormone profile (8). Similarly, after steroid discontinuation, an increase in the FT4 level was observed by Arai et al., but due to the small size of the study sample, some conclusions can be drawn from the data (7).

Nevertheless, due to improvements in the treatment and management of infants in the intensive care unit, the frequency of the incidence of THOP and delayed TSH in extra-preterm infants has declined. Limited data highlighted a significant difference in the thyroid status between preterm infants born with GA<28 weeks and those born more mature. In particular, controversies arise concerning the choice of treatment in preterm infants with BWs <1000 g or GA <28 weeks with incomplete development of the hypothalamus-pituitary-thyroid axis, as well as hypothalamic immaturity, therapeutic (24). It is not sufficient to distinguish THOP from central hypothyroidism, and the efficacy treatment with levothyroxine for persistent or profound TSH elevation or low FT4 levels in preterm infants remains unclear. Despite incomplete evidence, the current evidence seems to indicate that hypothyroxinemia in low birth weight infants should not be treated with levothyroxine as it fails to improve neurological outcomes (25, 26).

Most guidelines recommend levothyroxine treatment when the TSH serum level is >10 mU/l or the FT4 serum level is <10 pmol/l [0.8 ng/dl] at PNA 2-4 weeks (27, 28). A screening initiation of treatment should be implemented after persistent abnormal results measurements are taken 1-2 weeks apart (25, 29). Notably, preterm infants with BW <1000 g or born <28 weeks GA should undergo examinations on a case-by-case basis before levothyroxine treatment (27).

However, according to current knowledge, preterm infants with birth weight <1500 g or GA <32 weeks should be re-evaluated (30). Most researchers point out that after a primary screening test, rescreening of the measurement of the FT4 and TSH serum levels should be performed at the age of 2 and 4 weeks when a delayed TSH elevation is prominent. Some researchers recommend that a blood thyroid hormone test be performed at discharge (27).

This study is not without limitations. First, it was a single-center study with a relatively small sample size. Second, we did not evaluate the participants’ iodine status, but pregnant women in Poland received an iodine supplement according to the Polish Endocrine Society’s recommendation. Third, we did not obtain the expected results regarding the correlation between morbidity and FT4 and TSH values, which might have been related to the small sample size.

The study’s main strength was the identification of the GA-specific distribution of FT4 and TSH atPNA p2 weeks and PMA32 weeks, as well as the identification of the ultrasound thyroid volume value for preterm infants.

In summary, ultrasound thyroid imaging might provide valuable insight into evaluating questionable thyroid disorders. This study’s value cannot be denied as it allows the comparison of the thyroid gland’s ultrasound size with thyroid function as expressed by FT4 and TSH serum levels.

## Data Availability Statement

The original contributions presented in the study are included in the article/supplementary material. Further inquiries can be directed to the corresponding author.

## Ethics Statement

The studies involving human participants were reviewed and approved by Bioethical Committee of the Medical University of Warsaw (KB44/2019). Written informed consent to participate in this study was provided by the participants’ legal guardian/next of kin.

## Author Contributions

AM and RB conceptualized this study. AM wrote the first draft of this manuscript. RB critically reviewed the manuscript and accepted the final manuscript for submission. KK: She was recruiting patients. All authors read and approved the final version of the manuscript.

## Conflict of Interest

The authors declare that the research was conducted in the absence of any commercial or financial relationships that could be construed as a potential conflict of interest.

## Publisher’s Note

All claims expressed in this article are solely those of the authors and do not necessarily represent those of their affiliated organizations, or those of the publisher, the editors and the reviewers. Any product that may be evaluated in this article, or claim that may be made by its manufacturer, is not guaranteed or endorsed by the publisher.
